# Changes in Lutein Status Markers (Serum and Faecal Concentrations, Macular Pigment) in Response to a Lutein-Rich Fruit or Vegetable (Three Pieces/Day) Dietary Intervention in Normolipemic Subjects

**DOI:** 10.3390/nu13103614

**Published:** 2021-10-15

**Authors:** Begoña Olmedilla-Alonso, Elena Rodríguez-Rodríguez, Beatriz Beltrán-de-Miguel, Milagros Sánchez-Prieto, Rocío Estévez-Santiago

**Affiliations:** 1 Department of Metabolism and Nutrition, Institute of Food Science, Technology and Nutrition (ICTAN-CSIC), C/José Antonio Novais, 10, 28040 Madrid, Spain; msprieto@ictan.csic.es (M.S.-P.); Rocio.estevez@ufv.es (R.E.-S.); 2 Department of Chemistry in Pharmaceutical Sciences, Faculty of Pharmacy, Complutense University of Madrid (UCM), 28040 Madrid, Spain; elerodri@ucm.es; 3 Department of Nutrition and Food Science, Complutense University of Madrid (UCM), 28040 Madrid, Spain; beabel@farm.ucm.es; 4 Faculty of Experimental Science, Francisco de Vitoria University, 28223 Madrid, Spain

**Keywords:** lutein, xanthophylls, carotenoids, human serum, dietary intake, faeces, intervention study, macular pigment, contrast threshold, HDL-cholesterol

## Abstract

Lutein is mainly supplied by dietary fruit and vegetables, and they are commonly jointly assessed in observational and interventional studies. Lutein bioavailability and health benefits depend on the food matrix. This study aimed to assess the effect of dietary intervention with lutein-rich fruit or vegetables on lutein status markers, including serum and faecal concentrations (by high pressure liquid chromatography), dietary intake (24 h recalls ×3), and macular pigment optical density (MPOD) and contrast threshold (CT) as visual outcomes. Twenty-nine healthy normolipemic subjects, aged 45–65 y, consumed 1.8 mg lutein/day supplied from fruits (14 subjects, 500 g/day of oranges, kiwi and avocados) or vegetables (15 subjects, 180 g/day of green beans, pumpkin, and sweet corn) for four weeks. Serum lutein concentration increased by 37%. The effect of the food group intervention was statistically significant for serum lutein+zeaxanthin concentration (*p* = 0.049). Serum α- and β-carotene were influenced by food type (*p* = 0.008 and *p* = 0.005, respectively), but not by time. Serum lutein/HDL-cholesterol level increased by 29% (total sample, *p* = 0.008). Lutein+zeaxanthin/HDL-cholesterol increased, and the intervention time and food group eaten had an effect (*p* = 0.024 and *p* = 0.010, respectively) which was higher in the vegetable group. The MPOD did not show variations, nor did it correlate with CT. According to correlation matrixes, serum lutein was mainly related to lutein+zeaxanthin expressed in relation to lipids, and MPOD with the vegetable group. In faecal samples, only lutein levels increased (*p* = 0.012). This study shows that a relatively low amount of lutein, supplied by fruit or vegetables, can have different responses in correlated status markers, and that a longer intervention period is needed to increase the MPOD. Therefore, further study with larger sample sizes is needed on the different responses in the lutein status markers and on food types and consumption patterns in the diet, and when lutein in a “pharmacological dose” is not taken to reduce a specific risk.

## 1. Introduction

Lutein is a xanthophyll, an oxygenated carotenoid that is widely present in the human diet and is mainly supplied by the consumption of fruit and vegetables [1,2]. A high lutein intake, either through the diet or as a food supplement (alone or in combination with other food components), has several beneficial effects, especially on eye health (risk reduction of chronic disease and visual function improvement), in cognitive function [3] and in some measures of cardiovascular health, among other diseases [4]. However, there is still no consensus on the most appropriate dosage of daily lutein supplementation for each of the targets of its biological activities (in anti-inflammatory and antioxidant pathways, for instance) and for its role as a blue light filter, reducing phototoxic damage to photoreceptor cells [2,4]. Although supplementation with individual carotenoids may be beneficial for specific purposes, such as in the supplementation of lutein and zeaxanthin associated eye diseases (mainly on reducing the risk of progression of age-related macular degeneration) [2], there is still a need for data on carotenoid nutritional status, especially in populations consuming habitual diets. Preferably, this information should simultaneously assess several markers (serum markers and macular pigment optical density [MPOD], which are short and long-term markers, respectively) in well-defined and homogeneous populations, evaluating their bioavailability from different lutein sources and by analysing human faecal samples.

Serum lutein concentration is considered the best method for lutein nutritional status assessment. This concentration is expressed in relation to the blood lipid concentration to ease interpretation [5,6,7]. Instead, when nutritional status is approach by dietary methods, lutein and zeaxanthin levels are usually reported jointly because this is how they are still expressed in most of the food composition tables [8]. This occurs due to analytical constraints in the early food analysis by high-performance liquid chromatography (HPLC).

The plasma/serum carotenoid concentration is considered a marker of fruit and vegetable intake [2,9,10]. Although the health benefits of a high intake of these foods is well known, as the intake patterns of fruit and vegetables differ the effects of their consumption on health may also differ, and they should therefore be considered as different food groups [11]. Despite the fact that vegetables and green foods of plant origin are the major contributors to the dietary intake of lutein, the observed associations between fruit and vegetable intake and serum lutein concentrations are contradictory. While a lack of correlation between foods of green colour and serum/plasma lutein and zeaxanthin levels has been reported [12], some studies have found a higher correlation between serum lutein and zeaxanthin concentration and fruit intake instead [7]. Meanwhile, other studies show that it is the vegetable intake that is mostly associated with the serum/plasma lutein and zeaxanthin levels [13,14]. This discrepancy could be partially explained by the higher bioavailability of these compounds when ingested from fruit, where they are found in free and also in the more bioavailable esterified forms, as compared to vegetables [15,16,17]. In addition to this food-related factor affecting bioavailability (chemical forms present in foods), there are others such as the amount ingested (e.g., whole foods vs. food supplements), interactions with other dietary components that could have a positive (e.g., lipids) or negative (e.g., fibre) impact on carotenoid absorption. Host-related factors also influence bioavailability (e.g., sex, nutritional status, homeostatic control) [2]. On the other hand, regarding the relationship between MPOD and food intake, the strongest associations are found with fruit and red/orange foods (that are mainly fruits) in healthy adults (participants aged 45–65 years old, but not in the younger group) [18]. In addition, epidemiological studies assessing the role of fruit and vegetable intake on the risk of age-related macular degeneration describe a lower risk associated with a higher fruit intake but not from that of vegetables [19], as well as the protective role of a diet rich in green leafy vegetables, which are rich in lutein, against neovascular age-related macular degeneration [20].

Thus, in our study we hypothesised that a daily intake of lutein-rich fruit and vegetables, in a quantity compatible with a habitual diet, would increase the lutein serum concentration, and that this lutein serum response would be different depending on whether fruit or vegetables were eaten. We believe that this lutein increment could then impact the long-term lutein status marker MPOD. The aim of our study was to assess, in the Spanish population, the effect of a lutein-rich fruit and vegetable diet on lutein status biomarkers (serum concentrations, MPOD), the carotenoid content in faeces as a non-invasive approach to carotenoid bioavailability, and on the contrast threshold (CT) as a visual outcome in normolipemic subjects aged 45–65 years old.

## 2. Materials and Methods

### 2.1. Participants and Experimental Design

Twenty-nine volunteers (21 women) participated in a lutein-rich foods dietary intervention study and were divided into two groups (fruits-group, *n* = 14, and vegetable-group, *n* = 15) in two consecutive intervention periods because of the different seasonality of some of the foods supplied (March-April for the fruit group and June-July for the vegetable group) to consume a selection of fruits or vegetables daily during a four-week study. Thirty participants were recruited according to the following inclusion criteria: cholesterolaemia in the range of 3.9 to 6.5 mmol/L, body mass index (BMI) between 20 kg/m^2^ and 30 kg/m^2^, and mixed diet (no avoidance of any food groups). Volunteers were asked to report information on the following exclusion criteria: use of drugs or beverage/foods to control cholesterol levels, consumption of dietary supplements, surgery for myopia, cataracts or macular degeneration, and chronic diseases that can affect carotenoid or lipid metabolism. One participant dropped out after the beginning of the study. The same proportion of men and women was pursued but it was not reached because of the lower participation of men. The sample size was based on data obtained in a previous study in which 14 participants who had consumed 200 g broccoli/day (lutein content: 2400–3000 µg) for a week had a twofold increase in their lutein serum concentration [21]. In the present study the amount of lutein to be supplied was lower than in the aforementioned study, since our aim in the current study was to contribute at least the average total lutein and zeaxanthin dietary intake of the Spanish population (1235 µg lutein+zeaxanthin/day according to Estévez-Santiago et al. [1]), in contrast, the duration of the study was four times longer.

Participants were instructed to maintain a mixed diet (no avoidance of food groups) and to include certain fruit and vegetables in their habitual diets. The volunteers participated in the study during the spring and summer (April-May and June–July 2017, interventions with fruit and vegetables respectively). They underwent blood and faecal sampling, assessment of the MPOD and CT, and three 24-h diet recalls at baseline and at the end of the study period. Blood samples were collected after overnight fasting (at least 8 h) and at the same time as the first (out of three) 24-h diet recalls. Faecal samples were collected and kept until delivery to the laboratory on the same day of collection or frozen at −20 °C until delivery. Once received in the laboratory, they were kept at −80 °C, for a maximum of three months, under N_2_ atmosphere until lyophilisation. Samples were then placed in a freeze-drier for 48 h at −70 °C. Once lyophilized, they were returned to the freezer at −80 °C until extraction and analysis.

The Ethical Committee of Research with Drugs of the Hospital Universitario Puerta de Hierro-Majadahonda of Madrid, Spain (acta nº 03.17, dated 13 February 2017) and the Bioethic Subcommittee–Ethics Committee (CSIC) (dated 21 February 2017) approved this study. Written informed consent was obtained from all subjects.

#### Dietary Interventions

Lutein-rich foods were selected among those fruits and vegetables habitually consumed in Spain [22], in a quantity sufficient to supply at least the established mean dietary intake of Spanish adults (1235 µg lutein+zeaxanthin [total dietary intake]/day, 776 µg and 64 µg lutein+zeaxanthin/day from vegetables and fruits, respectively [1]. In an attempt to ensure a significant response in serum lutein concentration, we selected three fruits and three vegetables to supply an amount of lutein+zeaxanthin of approximately 1.8 mg/day, which could be considered compatible with a varied dietary intake.

The fruits and vegetables included in this study were avocados (Hass), kiwi (Oscar^®^, variety Hayward), oranges (Nave-Late), green beans, pumpkin (Cucurbita maxima, butternut) and sweet corn (canned). Average portion weights were 250 g of avocados, 100 g of kiwi and 150 g of oranges; 110 g of green beans, 50 g of pumpkin and 20 g of sweet corn. Lutein supplied by 500 g of fruit daily was 874 µg (plus 99 µg zeaxanthin/day from oranges) and that was supplied by 180 g of vegetables daily (green beans, pumpkin, corn): 954 µg (plus 54 µg of zeaxanthin/day from sweet corn). The intake of lamb’s lettuce (18–20 g/day) was included in both groups (fruit and vegetable), supplying 1002 µg lutein/day. Participants were instructed to eat kiwi and oranges as whole fruit and avocados alone or added to other dishes (i.e., as salad). Lutein and zeaxanthin intake was calculated using carotenoid content data reported elsewhere [23].

### 2.2. Analysis of Carotenoids in Serum and Faeces

Lutein, zeaxanthin, α-carotene, β-carotene and β-cryptoxanthin, (and lycopene only in faeces) concentrations were determined by HPLC using a system consisting of a model 600 pump, a Rheodyne injector and a 2998 photodiode array (PDA) detector (Waters, Milford, MA, USA) and a C30 YMC column (5 μm, 250 × 4.6 mm i.d.) (Waters, Wilmington, MA, USA) with a guard column (Aquapore ODS type RP-18). The mobile phase was methanol (MeOH) with 0.1% triethylamine (TEA)/methyl tert-butyl ether (MTBE), in a linear gradient from 95:5 to 70:30 in 25 min, to 35:65 in 25 min, to 95:5 in 10 min and maintained for 8 min. The flow rate was 1 mL min−1. The detection was performed at a wavelength of 450 nm. Identification was carried out by comparing the retention times with those of authentic standards and online UV-VIS spectra. Chromatogram data acquisition and processing Empower 2 software was used (Waters, Milford, MA, USA).

Lutein (xanthophyll from marigold), zeaxanthin, β-cryptoxanthin, lycopene, α-carotene, β-carotene, triethylamine, phosphate buffered saline (PBS), celite, potassium hydroxide, sodium chloride and butylated hydroxytoluene were obtained from Sigma-Aldrich (St. Louis, MO, USA). MTBE, MeOH, ethanol, dichloromethane (DCl), petroleum ether (PE) and diethyl ether (DE), were supplied by Análisis Vínicos (Ciudad Real, Spain). Anhydrous sodium sulphate and pyrogallic acid were supplied by Panreac (Barcelona, Spain).

#### 2.2.1. Analysis of Carotenoids and Lipids in Serum

Carotenoids extraction was performed on serum samples using a slight modification of a previously published method [24]. Briefly, 600 μL of serum was added to 600 μL of ethanol, vortexed and extracted twice with 1200 μL of hexane: dichloromethane (5:1) stabilized with 0.1 g/L butylated hydroxytoluene (BHT). The organic phases were pooled, evaporated to dryness under nitrogen atmosphere and reconstituted with 200 μL of a solution of MeOH: MTBE (1:1) and injected (50 μL) into the HPLC system.

Standard solutions were prepared from 1 mg of lutein and of zeaxanthin dissolved in 25 mL of tetrahydrofuran, with 0.01% BHT in each case. The E 1 cm 1% values used were: lutein, 2550 at 445 nm and zeaxanthin, 2540 at 450 nm. Working solutions were obtained from different volumes of the standard solutions dissolved in MeOH: MTBE (1:1 *v/v*).

Blood biochemical variables were analysed using the ADVIA Chemistry XPT System (Siemens Healthineers Spain). Total blood cholesterol was analyzed by the enzymatic assay and high-density lipoprotein (HDL) cholesterol, using a catalase assay kit. Low-density lipoproteins (LDL)-cholesterol level were calculated using the Friedewald equation [25].

#### 2.2.2. Analysis of Carotenoids in Faeces

Faecal carotenoid extraction and analysis have been detailed elsewhere [26]. Briefly, approximately 0.3 g of the lyophilized faecal sample was mixed with 10 mL of cool PBS 1x solution (pH = 7.4) and 2 mL of ethanol to hydrate the faeces and magnetically stirred for 20 min. Then, 20 mL of acetone was added and stirred for 4 min on a heat-free plate, the supernatant liquid was removed and the faecal samples were re-extracted with 10 mL of acetone and stirred for 4 min. Then, 10 mL of extraction solvent (DE:PE 1:1) were added, the sample was stirred for 2 min and centrifuged at 3500× *g* for 3 min. After recovering the coloured fraction, the residue was reextracted until colourless and the supernatants combined, and anhydrous sodium sulphate was added. The organic-coloured fractions were evaporated to dryness in a rotary evaporator, dissolved in 25 mL of MeOH:MTBE (50:50), and kept at −20 °C under nitrogen atmosphere until analysis.

To release hydrolysed xanthophyll fatty acid esters a saponification procedure was used [27]. Briefly, to 400 µL of the extracted carotenoids from the faeces, 400 µL of pyrogallic acid in ethanol (0.1 M), and KOH in MeOH (30%) were added and placed in an ultrasonic bath in the dark for 7 min. Then, 800 µL of distilled water and 1600 µL of extraction solvent were added, the mixture vortexed for 1 min, centrifuged for 3 min at 3500× *g*, and the organic phase was separated. This process was repeated twice. The organic phase collected was evaporated to dryness under nitrogen, reconstituted with 150 µL of MeOH:MTBE (50:50) and injected (50 µL) into the HPLC system.

Carotenoids were quantified by HPLC using calibration curves for lutein, zeaxanthin, β-cryptoxanthin, α-carotene, β-carotene and lycopene at four concentration levels. The concentrations of the carotenoids in the most diluted curve were: 0.19–1.5 ng/µL for lutein (R^2^ = 0.994), 0.15–1.20 ng/µL for zeaxanthin (R^2^ = 0.996), 0.11–0.90 ng/µL for α-carotene (R^2^ = 0.996), 0.21–2.10 ng/µL for β-carotene (R^2^ = 0.987)), 0.26–2.10 ng/µL for β-cryptoxanthin (R^2^ = 0.995) and 0.23–1.80 ng/µL for lycopene (R^2^ = 0.987). Three additional curves were used at higher concentrations. The concentrations of the carotenoids in the most concentrated curve were: 2.62–13.12 ng/µL for lutein (R^2^ = 0.992), 7.26–36.6 ng/µL for zeaxanthin (R^2^ = 0.990), 1.59–7.94 ng/µL for α-carotene (R^2^ = 0.996), 3.94–19.72 ng/µL for β-carotene (R^2^ = 0.999), 2.3–11.50 ng/µL for β-cryptoxanthin (R^2^ = 0.980) and 2.19–10.96 ng/µL for lycopene (R^2^ = 0.999).

### 2.3. Dietary Intake Assessment

Three 24-h diet recalls were used to assess the recent dietary intake at baseline and at the end of the intervention. These recalls were carried out within a period of seven days, one of which coincided with a weekend or holiday. The participants recalled their first report face-to-face with a specialized interviewer, the same person who subsequently made the two recalls by telephone. The amounts consumed were estimated in units (fruits), portions or household servings [28] and the corresponding food intake in grams per day were used to determine the daily intake of lutein, zeaxanthin and other carotenoids using a database [23] included in a software application for the calculation of carotenoid dietary intake [29].

To assess the total fat, saturated fatty acids (SFA), monounsaturated fatty acids (MUFA), polyunsaturated fatty acids (PUFA), cholesterol and energy intake, we used the DIAL© software [30], which includes a food composition table.

#### Database of Carotenoid Contents in Spanish Fruits and Vegetables

This database contains data on the individual major dietary carotenoids present in foods and human serum, which were previously generated by HPLC [23,31,32]. Foods analyzed were mainly fruit and vegetables, as these are the major contributors to dietary carotenoid intake and reported data correspond to the edible portion of foods grown and commercialized in Spain. Carotenoid concentration data corresponded to raw foods but also to cooked foods when they were habitually consumed cooked. Although 124 food types are included in the database, it should be noted that the information for 27 of them was taken from the literature as the combined lutein and zeaxanthin content (e.g., eggs, olive oil) and therefore the total dietary intake of lutein and zeaxanthin was considered as the sum of the two.

### 2.4. Body Fat Composition Assessment

Body fat composition (%) was calculated with an InnerScan©, Segmental Body Composition monitor (Tanita©, model BC-601), using the bioelectrical impedance analysis technique.

### 2.5. Assessment of the MPOD

MPOD was assessed using an MPS 9000 desktop device (Macular Pigment Screener; Elektron PLC, Cambridge, UK), using the principles of heterochromatic flicker photometry (described by van der Veen et al. [33]). The test consisted of two stages for central and peripheral viewing, and the subjects were required to press a response button whenever the flicker was detected. The subjects started by fixating the central stimulus, a 1-degree central target. The flicker rate was initially set at 60 Hz and then gradually reduced at a rate of 6 Hz s−1. The observer then fixated on red 2° diameter target placed 8° eccentrically and a second set of data was recorded for peripheral viewing [34]. The process was repeated for a series of green-blue luminance ratios. The MPOD was measured in density units (du) and ranged from 0 to1.

### 2.6. Visual Contrast Sensitivity and Contrast Threshold

The contrast sensitivity was measured using its inverse, CT. The CT was determined using an automated strategy, set for six sizes of annular stimuli with diameters ranging from 6.3° to 0.7° of the visual angle, with and without glare light conditions. There were 12 levels of CT, ranging from 0.01 to 0.45. The CGT-1000 Contrast Glaretester (Takagi, Japan) was used. Each subject was tested monocularly for CT, once with each eye and with spectacle correction when necessary. The lower the CT, the higher the contrast sensitivity level at which the subject was able to detect each spatial frequency.

The luminance of the background on which the stimulus was presented was 10 cd/m^2^. The specifications selected for the presentation of the stimulus were: presentation duration, 0.2 s; presentation interval, 2 s; test luminance with glare of 40,000 cd/m^2^; test distance, 350 mm. The device had eight glare sources arranged around the stimulus that were activated automatically to assess the CT with a simultaneous glare.

### 2.7. Statistical Analysis

Data are expressed as the mean ± standard deviation (SD) and median. The mean values were compared using parametric (paired t-test) and, when data did not follow a normal distribution (assessed by the Kolmogorov-Smirnov test), by nonparametric tests (Mann-Whitney U test, Wilcoxon). The analysis of the relationship between the serum variables, MPOD and CT was carried out using a Pearson matrix correlation or Spearman for the CT variables. All reported *p*-values were based on a two-sided test and compared to a significance level of 0.05. SPSS Statistics Editor (IBM SPSS Statistics, v.27) was used for all statistical calculations.

To assess the evolution of the variables analyzed (differences between the baseline and final concentrations), a paired t-test was used (with the total sample) and a mixed model of repeated measurements with the time factor (baseline–final) as repeated measurements and the fruit and vegetable group and the time as the main effect. A Paired ANOVA was used to assess the effect of the dietary intervention with fruit or vegetables. The effect of the fruit or vegetable intervention group at each point was assessed with a t-test for each variable at each point with the group assigned (fruit or vegetable) as a factor.

General linear model (GLM) analysis using the variables in serum and diet (lutein and zeaxanthin in serum and dietary intake, lipids in serum and group of fruit/vegetables) and MPOD as dependent variable, at the baseline and at the end of the study, showed as a unique variable in the model, the fruit and vegetable group, and thus resulted to be insufficient to be considered as a predictive model. Thus, the factor analyses were performed (extraction method: principal component analysis; rotation method: Varimax rotation with Kaiser normalization) to examine the following sets of serum variables: lutein in serum and (1) fruit and vegetable group, lutein, zeaxanthin and lutein+zeaxanthin, lutein/HDL-cholesterol, lutein+zeaxanthin/HDL-cholesterol and (2) cholesterol, LDL-cholesterol and HDL-cholesterol. Variables used to asses MPOD: the fruit and vegetable groups, the lutein+zeaxanthin intake, the serum concentrations of lutein, lutein+zeaxanthin, lutein/HDL-cholesterol, lutein+zeaxanthin/HDL-cholesterol and HDL-cholesterol. The objective was to identify multivariable relationships by taking each correlation matrix. The results are presented as plots of component loadings. Each point is connected to the origin, and the angles between segments express the measurement of the correlation (angles narrower than 90° indicate positive correlation, wider angles indicate negative correlation).

## 3. Results

The characteristics of the participants are listed in Table 1. There were no differences in the age, cholesterolaemia, BMI and body fat between the participants of the different groups. The number of men and women in each group was not the same due to the lower interest of men in the study in general and in the vegetable intervention in particular.

The quantity of lutein supplied to each group is shown in Table 2. The daily lutein supply was practically the same in both groups (3.07 µmoL/day in the fruit group and 3.21 µmol/day in the vegetable group); however, the daily weight of fruit (500 g) and vegetables (180 g) needed to supply the intake were different. The total dietary intake of lutein plus zeaxanthin, fat, SFA, MUFA, PUFA and cholesterol at baseline and at the end of the study is shown in Table 3. There was a significant increase in lutein plus zeaxanthin in the total sample (*p* = 0.0001) and in each of the intervention groups (fruit group *p* = 0.001 and vegetable group, *p* = 0.020). There was no statistically significant difference between the groups (fruit and vegetables) at the beginning and at the end of the study. No differences were found in the total fat intake or in the intake of SFA, MUFA, PUFA or cholesterol between baseline and the end of the study in the fruit and vegetable groups, the lone exception being MUFA intake in the fruit group (*p* < 0.01). In the total sample, a significant increase in the total lipids (*p* = 0.028) and in MUFA (*p* = 0.014) intake was obtained at the end of the study. There was no difference in energy intake at the beginning and at the end of the study (2193 ± 628 and 2216 ± 655 Kcal, respectively).

Serum lutein concentration and that of other major carotenoids in the blood (only those supplied with the foods used in the study were considered: zeaxanthin, β-cryptoxanthin, α-carotene and β-carotene), cholesterol (total, HDL and LDL) and MPOD at baseline and at the end of the study are shown in Table 4. After a daily lutein-rich food intake supplying around 1.8 mg lutein/day, serum lutein concentration increased by 37.1% (*p* = 0.004) and that of lutein+zeaxanthin by 21.5% (*p* = 0.012) overall. This lutein increase was greater in the fruit-group than in the vegetable-group (52% vs. 23%, *p* = 0.059). There was a significant increase in serum lutein+zeaxanthin concentration (*p* = 0.049) with the food group interventions (fruit or vegetable) but no effect on effect on lutein concentration alone (*p* = 0.064). At baseline, serum lutein concentration (and that of lutein+zeaxanthin) in the vegetable-group was higher than that in the fruit group (*p* = 0.021); however, there were no differences in the concentrations reached at the end of the study between groups.

The serum concentrations of the other carotenoids showed statistically significant variations at the end of the study, except for zeaxanthin and β-cryptoxanthin. The serum α- carotene and β-carotene concentrations were influenced by the food supplied (*p* = 0.008 and *p* = 0.005, respectively), being higher in the vegetable than in the fruit group (*p* = 0.028 and 0.038 for α-carotene and β-carotene, respectively) and was not modified by the time.

In the total sample, HDL-cholesterol, the major lipoprotein transporter of lutein and zeaxanthin, LDL-cholesterol and cholesterol did not change significantly at the end of the study. There were no variations in these lipids neither in the total sample nor in the two groups (fruit and vegetables); however, considering paired data, HDL-cholesterol increased in the fruit group (*p* = 0.031) and decreased in the vegetable group (*p* = 0.027). When lutein was expressed in relation to lipids, the lutein/HDL-cholesterol increased 29% in the total sample (*p* = 0.008) and in each of the groups (*p* = 0.031 in fruit group and *p* = 0.041 in vegetable group) and, considering both intervention groups, this increment was higher in the vegetable group (*p* = 0.010). There was also an increase in the lutein+zeaxanthin/ HDL-cholesterol (*p* = 0.000 in total sample and, *p* = 0.002 in the fruit group and *p* = 0.041 in the vegetable group), and the time and the type of intervention group had an effect (*p* = 0.024 and *p* = 0.010, respectively). These increments were higher in the vegetable group than in the fruit group (*p* = 0.045 and *p* = 0.048, respectively). Both ratios showed differences at baseline between the two intervention groups (*p* = 0.028 and *p* = 0.034, fruit and vegetable groups, respectively), but not at the end of the study. Serum lutein +zeaxanthin/HDL-cholesterol showed an inverse correlation with lutein+zeaxanthin intake at baseline (rho = −0.430, *p* = 0.020) and with HDL-cholesterol at the end of the study (rho = −0.426, *p* = 0.021).

MPOD did not show variation at the end of the intervention study, in the total sample, or in the two groups. However, the MPOD showed differences between intervention groups at baseline (*p* = 0.032) and at the end (*p* = 0.004) of the study, which was higher in the vegetable group than in the fruit group. MPOD did not show any significant correlations, either at baseline or at the end of the study, with any of the variables analysed in the serum and in dietary intake, except for HDL-cholesterol (rho: −0.378, *p* = 0.039) in the vegetable-group at the end of the study.

**Figure 1:**
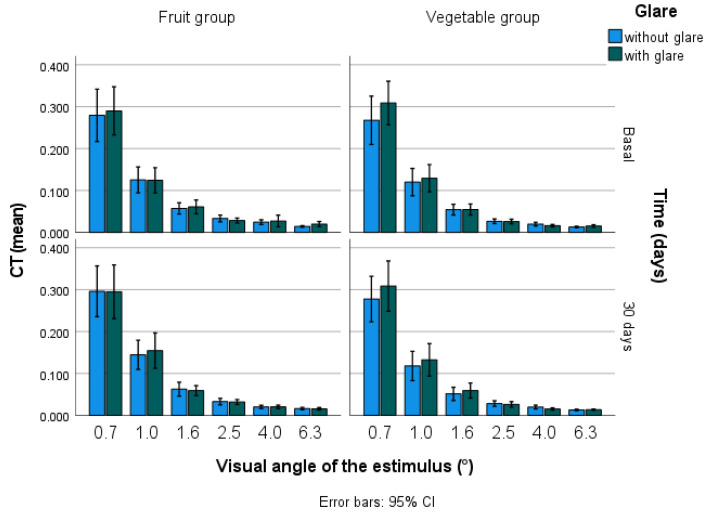
Contrast threshold at different degrees of visual angle, without and with glare, at the beginning and the end of the study.

CT, with and without glare, as measured as the final outcome of the MPOD (Table S2), did not show any correlation with the MPOD at the end of the study, except with a 0.7° visual angle, with glare (rho= 0.306 *p* = 0.20). There were differences in CT, with and without glare, at all of the visual angles, between the beginning and the end of the study (*p* < 0.001) (Figure 1). Between the intervention groups, there were differences in the CT data, with glare, at 6.3 (*p* = 0.05) and 4.0 (*p* = 0.04).

**Figure 2:**
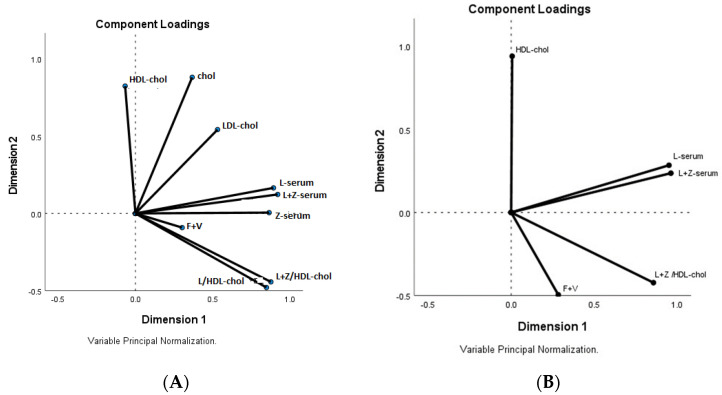
Factor analysis loading plot. Two principal components. (**A**) Serum lutein, fruit and vegetable groups, zeaxanthin and lutein+zeaxanthin, lutein/HDL-cholesterol, lutein+zeaxanthin/HDL- cholesterol, cholesterol, HDL-cholesterol, and LDL-cholesterol. (**B**) without cholesterol and LDL-cholesterol.

In the analysis of the correlation matrix for lutein and zeaxanthin concentrations, cholesterol (total, HDL- and LDL-) explained the 73.43% of the variance and showed that the fruit and vegetable group were not related to any of the variables analysed in the serum, despite that both saturated in the same factor 1. Cholesterol (total, LDL- and HDL) saturated in factor 2 (Figure 2). Lutein and zeaxanthin, the variables obtained when associated with lipids and the fruit and vegetable groups, saturated in factor 1 and cholesterol (total, HDL-) saturated in factor 2. Lutein in serum was mainly associated with lutein and zeaxanthin expressed in relation to lipids.

**Figure 3:**
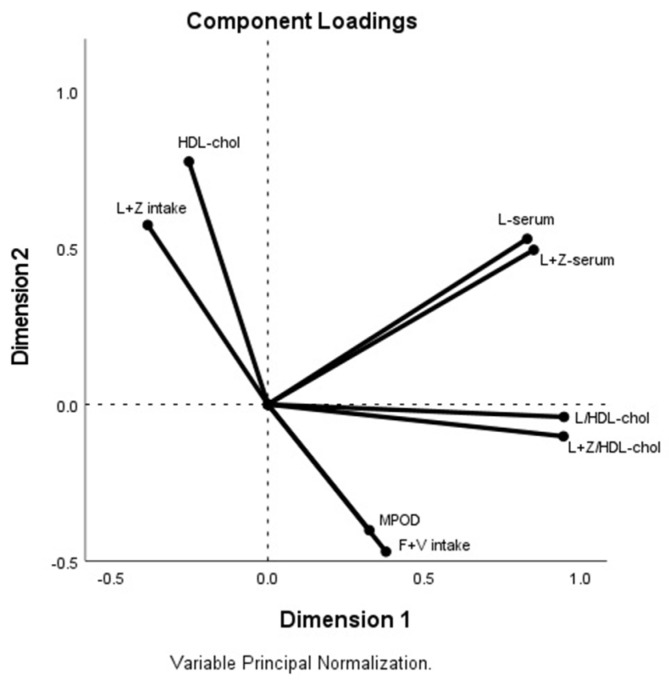
Factor analysis loading plot. Two principal components. MPOD, fruit and vegetable groups, lutein+zeaxanthin intake, serum lutein, lutein+zeaxanthin, lutein/HDL-cholesterol, lutein+zeaxanthin/HDL-cholesterol and HDL-cholesterol concentrations at the end of the study.

Figure 3 shows the associations among variables considering short and long-term lutein status markers (serum lutein+zeaxanthin concentration and MPOD), blood lipids, and the fruit or vegetable groups. Factorial analysis explained the 69.8% and 68.7% of the variance at the beginning and end of the study, respectively. MPOD showed the strongest and most positive association with the fruit/vegetable groups; the highest values of MPOD were related to the vegetable group (0.255, *p* = 0.054 and 0.363, *p* = 0.005 at the initial and final stages, respectively), followed by lutein/HDL (initial value 0.167, *p* = 0.10 and final value: 0.240, *p* = 0.070) and lutein+zeaxanthin/HDL-cholesterol (initial value 0.132, *p* = 0.325 and, final value: 0.237, *p* = 0.074). There were two other blocks of variables weakly associated with MPOD, lutein and lutein+zeaxanthin; these were inversely associated with lutein+zeaxanthin intake and HDL-cholesterol.

**Figure 4:**
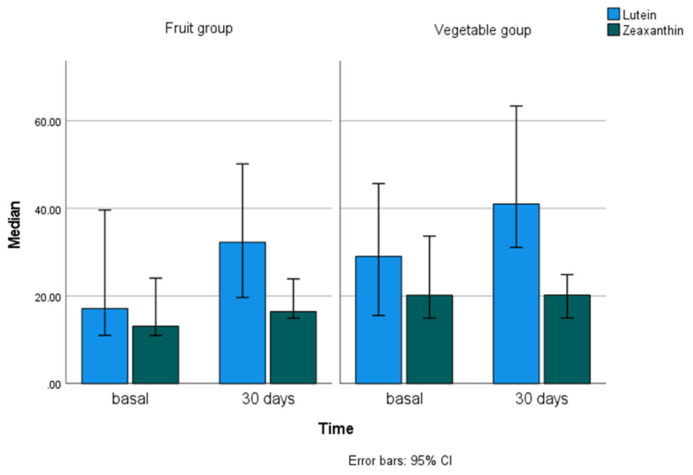
Lutein and zeaxanthin concentrations (µg/g dry weight) in the faeces at the beginning and end of the study in each group.

Finally, the carotenoid concentrations in the faeces are shown in Table 5. These carotenoids quantified in faeces were lutein, zeaxanthin, β-cryptoxanthin, α-carotene, β-carotene and lycopene. At baseline, the highest concentration in faeces corresponded to β-carotene, followed by lycopene and lutein, and corresponded to the ones consumed in the greatest amounts by participants (Table S1). At lower concentrations were zeaxanthin, α-carotene and β-cryptoxanthin. Differences in faecal lutein concentration were only found between the baseline and final values of the study in the total sample (*p* = 0.012) and in the vegetable group (*p* = 0.056), but not in the fruit group. No differences were found for any of the other carotenoids analysed in the faecal samples. Considering the two intervention groups, there were differences, at the end of the study, in the concentrations of β-cryptoxanthin (*p* = 0.027), α-carotene (*p* < 0.001) and lycopene (*p* = 0.001 and also at baseline: *p* = 0.004). Figure 4 shows lutein and zeaxanthin concentrations in the faeces at the beginning and end of the study in each group. Only two of the carotenoids analysed in faeces, lycopene and β-cryptoxanthin, showed significant correlation with their respective dietary intakes, lycopene at baseline and at the end of the study (rho = 0.578, *p* = 0.001) and β-cryptoxanthin at baseline (rho = 0.427, *p* = 0.021).

## 4. Discussion

In this study, normolipemic subjects ate foods supplying 1.8 mg lutein/day, from fruit or vegetables, to their habitual diets in order to observe potential differences due to food matrices, as correlated with lutein bioavailability [2,16,17]. Although there are numerous studies including dietary intervention with fruit and vegetables to achieve different aims, these foods are usually supplied jointly [11]. However, different effects of fruits and vegetables have been described in several studies, such as those showing a lower risk of age-related macular degeneration associated with a higher intake of fruit but not of vegetables [19], associations of lutein/zeaxanthin concentrations and fruit intake (but not that of vegetables) in relation to insulin-like growth factor binding proteins [35] and associations with lutein status markers [7,11,14,18]. In the present study, the participants received a lutein concentration higher than that consumed by the Spanish adult population from fruit and vegetables, 0.8 mg/day (mean) [1] and 0.5 mg lutein/day (median) eaten by subjects with similar characteristics as the ones in this study [7], but lower than the quantity used in other intervention studies (e.g., 2.4–3 mg/d from broccoli in Granado et al. [21]) and in studies using lutein as a supplement (5–20 mg/d associated with MPOD) [36].

The fruit and vegetable weights to be consumed daily should have been similar in order to have the same impact on the habitual diet. However, because of the higher lutein density in vegetables than in fruits, this was not feasible, and the amount of fruit consumed was significantly higher than that of vegetables (500 g and 180 g, respectively). In any case, these amounts could be considered compatible with a varied dietary intake, since a daily intake of 400–600 g fruit and vegetables is recommended for the general population [37,38]. Our findings indicated that the dietary intervention lead to an increment in the serum lutein concentration (37%) that is proportional to that reported in a broccoli intervention study (50%) in a group of normolipemic younger subjects (20–35y), in which the lutein supplied was in the range of 2.4–3 mg/day [21]. The lutein and lutein+zeaxanthin increments were independent of the type of food, and no differences were found between the fruit or vegetable intake, although the serum response in the fruit group was higher than that in the vegetable group. This higher response can be explained by the daily intake of avocado in the fruit group owing to the fact that it is rich in MUFA, which in turn enhances lutein bioavailability, and has also been associated with increases in HDL-cholesterol [39], as also occurred in our fruit group. A trend in the response between fruit and vegetable groups was seen, but statistical significance was not achieved, most likely because of the high variability in the participant’s serum and dietary intake responses within each intervention group, and also to the effect of the different ways that fruits and vegetables were consumed, which could have led to varying lutein bioavailability and thus different response levels in the status markers. Vegetables supplied were consumed cooked and mixed with oil or seasoning which tend to enhanced carotenoid bioavailability [2]. However, the fruit supplied was consumed raw and perhaps between meals (oranges and kiwi) but avocados were mainly consumed together with other foods and sometimes with oil or seasoning added.

The lutein-rich fruit and vegetables used in this intervention study supplied other carotenoids such as β-cryptoxanthin by oranges and, at lower concentrations, α-carotene (oranges and avocados), β-carotene (kiwi, avocados and oranges) and zeaxanthin (oranges) in the fruit group. In the vegetables group, α-carotene and β-carotene (green beans, sweet corn and pumpkins) were supplied at higher concentrations than in the fruit group, and zeaxanthin was supplied by sweet corn. In both groups, lamb’s lettuce contained a large amount of β-carotene. However, only the serum α-carotene and β-carotene concentrations increased significantly. A lutein dietary intervention study with egg yolks that supplied 1.9 mg lutein/day led to the same serum lutein concentration as our study, and did not change the other main serum carotenoids [40].

We found that the lutein-rich fruit and vegetable intake led to an increase in the serum lutein (37% in total sample) and, consequently, to a lutein/HDL-cholesterol and lutein+zeaxanthin/HDL-cholesterol increment in the total sample and in each of the groups. This could have been due to a decrease in HDL-cholesterol, but this was not the case in our study. The increase in serum carotenoid-HDL concentration (29%) was higher than that obtained with an egg yolk dietary intervention (five weeks) in subjects of similar age (20%). However, in the latter example, the participants were taking cholesterol-lowering statins, which could have explained the increment found [40].

The long-term lutein biomarker, MPOD, was not modified in our study. The amount of lutein supplied, the length of the study and the lutein status (blood concentration and MPOD) are crucial in obtaining a response and, in general, higher amounts of lutein (mainly as food supplements that are more bioavailable), longer intervention periods and a low lutein status are key aspects in increasing MPOD [33], although an MPOD increment (31%) has been described with a dietary intervention with egg yolks (five weeks, 1.9 mg lutein/day) [40], probably due to the higher bioavailability of lutein from eggs and because of the simultaneous presence of zeaxanthin in eggs. The MPOD values obtained in our study are similar to those found in apparently healthy subjects that have similar characteristics, as previously reported by our group [7,41]. As a functional outcome of the MPOD, we determined the CT and found no relevant correlations. However, an observational study with subjects of similar characteristics found that MPOD showed strong association with CT, mainly at medium and smaller visual angle degrees, which corresponded to medium and high spatial frequencies [41]. These spatial frequencies are the ones that decline with age, leading to a reduction in visual acuity [42].

Serum lutein concentration is considered a marker of fruit and vegetable intake [9] and, in a previous study with subjects of the same age as our study group, serum lutein and zeaxanthin concentrations were more strongly associated with fruit intake (rho = 0.318) than with vegetable intake (rho = 0.255). However, in our study, fruit and vegetable groups were not related to any of the variables analysed in serum, which could be due to the small sample size and the high variability in the results among subjects. On the other hand, the MPOD showed a strong association with the fruit or vegetable groups, and the highest associations were between the MPOD and vegetable group. This is in contrast to a previous study in which MPOD was associated with fruit, but not vegetable intake [1]. In this respect, the influence of potential interactions among carotenoids supplied along with lutein in our intervention study cannot be ruled out, mainly because of the high concentration of β-carotene supplied [43].

The carotenoid concentration in faeces has been used as a non-invasive approach to evaluate carotenoid bioavailability [44,45]. The highest concentration in faeces in our study corresponded to β-carotene and lycopene, which are also generally more abundant in the human diet and blood [2,46,47]. The carotenoid concentration in this study was higher than that found in similar volunteers (*n* = 101) in a previous study [26], and higher (more than two times the β-carotene and lycopene levels) than those described in young women (*n* = 7) [48]. This can be explained by a higher intake of fruits and vegetables or, less likely, because a 24-h faecal sample was not collected and the fraction delivered to the laboratory may have had, by chance, a higher concentration of carotenoids. On the other hand, the low carotenoid bioavailability from fruits and vegetables (ca. 10–40%, [2,26] could also partially explain the concentrations in faeces obtained in this study, in which both non-green and green fruits and vegetables were supplied. The bioaccessibility of lutein from non-green vegetables is higher than that from green vegetables, even though they are the most abundant sources of lutein [49,50], which could explain the similarity in lutein elimination between the two intervention groups. In this sense, there was an increase in the lutein in faeces, but only in the vegetable group, which could have been due to the higher serum lutein concentration in the vegetable group (28 vs. 38 µg/dL, *p* < 0.05), a better lutein status, and thus, a lower lutein absorption and, therefore, a greater faecal elimination. This inverse predictive value of serum carotenoid concentration changes has been described previously [51,52], although the mechanisms by which the baseline status of non-provitamin A carotenoids may affect their absorption are unknown [53].

Our data is valuable since intervention studies with foods rich in specific carotenoids that are compatible with the habitual diet of normolipidemic adults are scarce in healthy volunteers. There are similar published studies, but in individuals taking cholesterol-lowering statins [40] and in individuals with low fruit and vegetable intake [54]. We evaluated MPOD and CT to infer the optical implications of our approach. Other studies have also attempted to reduce the risk of ocular diseases and improve visual function, but this was done using higher lutein and zeaxanthin concentrations as food supplements [55,56]. The major limitations of the study were the small sample size given the high variability found in the carotenoid concentrations among these volunteers. This restricted findings with significant differences in the serum and MPOD response between the two interventions groups. Likewise, another limitation was the lack of homogeneity of the faecal samples and the fact that each given sample ids not represent of a 24-h faecal excretion, nor can an adequate correlation be drawn with any other biomarker. The strengths of this study were the design, with a relatively low amount of lutein supplied by fruit and vegetables in the context of the habitual diet. Furthermore, we chose normolipemic subjects in an age range in which lutein status improvement can benefit their health and, finally, we carried out a simultaneous assessment of cholesterolaemia and four markers of lutein status (diet, serum, faeces, and MPOD).

## 5. Conclusions

This intervention study shows that an increase in lutein intake (1.8 mg/day), achieved through marginal dietary modifications using fruit or vegetables, can elicit different responses in correlated status markers. In this study, short-term biomarkers changed but not the long-term biomarker MPOD, which can only be expected to increase following a longer period of dietary intervention. Moreover, with this type of dietary intervention, the intake of other bioactive compounds and nutrients contained in these fruits and vegetables was also beneficial and may also play a role, as lutein does, in the visual and cognitive functions. Furthermore, this was done with minimal dietary modifications, as this would minimize imbalances among bioactive compounds/nutrients in the overall diet (i.e., interactions between lutein and β-carotene in absorption are well known).

These results point to the need to delve deeper into the different macular pigment responses to the intake of fruits or vegetables in the context of people’s general everyday diet in larger samples, and when lutein in “pharmacological doses” is not taken to reduce a specific risk. Special attention should be paid to subjects’ lutein status and lipid profile, as well as to the way in which foods are eaten, as many host-related and dietary factors can affect its bioavailability.

## Figures and Tables

**Table 1:** Characteristics of the participants in the intervention study.

	Total Sample (*n* = 29)	Fruit Group (*n* = 14)	Vegetable Group (*n* = 15)
Age	55.6 ± 4.9	57.0 ± 4.4	54.3 ± 5.2
Sex (M/F)	8/21	5/9	3/12
Body mass index (kg/m^2^)	24.2 ± 2.8	24.7 ± 2.8	23.7 ± 2.8
Body fat (%)	30.1 ± 6.5	29.3 ± 7.2	30.8 ± 5.9
Cholesterol	5.30 ± 0.70	5.16 ± 0.76	5.42 ± 0.64
HDL-cholesterol	1.70 ± 0.38	1.68 ± 0.39	1.72 ± 0.39
LDL-cholesterol	3.18 ± 0.48	3.02 ± 0.76	3.33 ± 0.52

M: male, F: female. LDL: low density lipoprotein; HDL: high density lipoprotein.

**Table 2:** Lutein (µmol/day) was supplied by fruit and vegetable addition (g/day) to the diet.

	Lutein	Weight
Fruits ^1^	1.54	500
Vegetables ^2^	1.68	180
Fruits plus lamb’s lettuce	3.07	520
Vegetables plus lamb’s lettuce	3.21	200

^1^ avocados (250 g/day), kiwi (100 g/day), oranges (150 g/day). ^2^ green beans (110 g/day), pumpkin (50 g/day), sweet corn (20 g/day).

**Table 3:** Lutein and zeaxanthin (µmol/day), fat, SFA, MUFA, PUFA and cholesterol (g/day) total dietary intake expressed as mean ± SD [median].

	Total Sample	Fruit Group	Vegetable Group (*n* = 15)
	(*n* = 29)	(*n* = 14)	
Lutein+zeaxanthin			
Basal	2.26 ^a^ ± 1.31 [1.88]	2.16 ^a^ ± 1.03	2.35 ^a^ ± 1.56
Final	4.94 ^b^ ± 3.16 [3.80]	6.06 ^b^ ± 3.88	3.90 ^b^ ± 1.89
Fat intake			
Basal	96.4 ^a^ ± 34.7	108.0 ± 39.7	85.5 ± 26.1
Final	112.3 ^b^ ± 42.7	134.4 ± 49.7	84.7 ± 27.6
SFA			
Basal	29.2 ± 10.4	32.9 ± 10.7	25.6 ± 9.1
Final	28.2 ± 10.5	32.3 ± 10.8	24.3 ± 9.0
MUFA			
Basal	42.9 ^a^ ± 20.1	49.6 ^a^ ± 24.4	36.6 ± 13.1
Final	54.8 ^b^ ± 22.8	72.6 ^b^ ± 16.2	38.1 ± 13.5
PUFA			
Basal	16.0 ± 5.8	17.1 ± 6.5	15.0 ± 5.2
Final	17.5 ± 8.9	20.5 ± 10.3	14.6 ± 6.6
Cholesterol			
Basal	306 ± 156	322 ± 130	291 ± 176
Final	294 ± 133	313 ± 141	277 ± 128

Different superscripts indicate significant difference between basal and final data.

**Table 4:** Lutein and other major carotenoid concentrations (µmol/L) and cholesterol (total, HDL-, LDL-) (mmol/L) in serum and MPOD (d.u.) at baseline and after four weeks of intervention study.

	Total Sample	Fruit Group	Vegetable Group
	(*n* = 29)	(*n* = 14)	(*n* = 15)
Lutein			
Basal	0.60 ± 0.21 ^a^	0.50 ± 0.16 ^a A^	0.68 ± 0.22 ^a B^
Final	0.77 ± 0.25 ^b^	0.75 ± 0.25 ^b^	0.80 ± 0.26 ^b^
Zeaxanthin			
Basal	0.13 ± 0.01	0.11 ± 0.05	0.15 ± 0.07
Final	0.13 ± 0.06	0.12 ± 0.04	0.14 ± 0.07
Lutein+zeaxanthin			
Basal	0.72 ± 0.26 ^a^	0.61 ± 0.19 ^a A^	0.83 ± 0.28 ^B^
Final	0.91 ± 0.30 ^b^	0.87 ± 0.27 ^b^	0.94 ± 0.32
β-cryptoxanthin			
Basal	0.76 ± 0.62	0.74 ± 0.44	0.78 ± 0.76
Final	0.68 ± 0.53	0.70 ± 0.29	0.66 ± 0.69
α-carotene			
Basal	0.16 ± 0.09 ^a^	0.13 ± 0.08	0.18 ± 0.09
Final	0.21 ± 0.18 ^b^	0.14 ± 0.07 ^A^	0.28 ± 0.22 ^B^
β-carotene			
Basal	1.17 ± 0.82 ^a^	0.82 ± 0.43 ^a A^	1.50 ± 0.97 ^B^
Final	1.44 ± 1.03 ^b^	1.08 ± 0.67 ^b A^	1.78 ± 1.20 ^B^
Cholesterol			
Basal	5.3 ± 0.7	5.2 ± 0.8	5.4 ± 0.6
Final	5.2 ± 0.6	5.2 ± 0.7	5.3 ± 0.6
HDL-cholesterol			
Basal	1.7 ± 0.4	1.7 ± 0.4 ^a^	1.7 ± 0.4 ^a^
Final	1.7 ± 0.4	1.8 ± 0.4 ^b^	1.6 ± 0.4 ^b^
LDL-cholesterol			
Basal	3.2 ± 0.5	3.0 ± 0.8	3.3 ± 0.5
Final	3.1 ± 0.5	3.0 ± 0.5	3.3 ± 0.4
Lutein/HDL-cholesterol (µg/mg)			
Basal	0.52 ± 0.21 ^a^	0.43 ± 0.11 ^a A^	0.60 ± 0.25 ^a B^
Final	0.67 ± 0.24 ^b^	0.60 ± 0.15 ^b A^	0.74 ± 0.29 ^b B^
Lutein+zeaxanthin/HDL-cholesterol (µg/mg)			
Basal	0.63 ± 0.26 ^a^	0.53 ± 0.14 ^a A^	0.73 ± 0.31 ^a B^
Final	0.79 ± 0.29 ^b^	0.70 ± 0.16 ^b A^	0.87 ± 0.36 ^b B^
MPOD			
Basal	0.34 ± 0.12	0.31 ± 0.12 ^A^	0.37 ± 0.12 ^B^
Final	0.33 ± 0.13	0.28 ± 0.10 ^A^	0.38 ± 0.14 ^B^

Superscript letters indicate significant differences between basal and final concentrations (^a^ columns) and between groups (^A^ rows). D.U.: density units; LDL: low density lipoprotein; HDL: high density lipoprotein; MPOD: macular pigment optical density.

**Table 5:** Lutein and other major carotenoid concentrations in faeces (µmol/day, dry weight) at baseline and after four weeks of intervention study. Mean ± SD [median].

	Total Sample (*n* = 29)	Fruit Group (*n* = 14)	Vegetable Group (*n* = 15)
Lutein			
Basal	0.051 ± 0.035 [0.043] ^a^	0.050 ± 0.039 [0.030]	0.055 ± 0.033 [0.051] ^a^
Final	0.078 ± 0.069 [0.061] ^b^	0.059 ± 0.027 [0.057]	0.095 ± 0.090 [0.072] ^b^
Zeaxanthin			
Basal	0.034 ± 0.019 [0.033]	0.030 ± 0.017 [0.023]	0.040 ± 0.020 [0.036]
Final	0.040 ± 0.030 [0.032]	0.036 ± 0.018 [0.029]	0.045 ± 0.038 [0.036]
β-cryptoxanthin			
Basal	0.010 ± 0.021 [0.000]	0.011 ± 0.020 [0.000]	0.009 ± 0.022 [0.000]
Final	0.007 ± 0.013 [0.003]	0.007 ± 0.006 [0.005] ^A^	0.008 ± 0.018 [0.000] ^B^
α-carotene			
Basal	0.027 ± 0.047 [0.003]	0.017 ± 0.033 [0.000]	0.036 ± 0.056 [0.012]
Final	0.030 ± 0.026 [0.017]	0.013 ± 0.034 [0.000]^A^	0.047 ± 0.048 [0.036] ^B^
β-carotene			
Basal	0.146 ± 0.129 [0.088]	0.151 ± 0.158 [0.062]	0.142 ± 0.101 [0.108]
Final	0.147 ± 0.120 [0.102]	0.107 ± 0.107 [0.061]	0.184 ± 0.123 [0.193]
Lycopene			
Basal	0.118 ± 0.133 [0.064]	0.051 ± 0.072 [0.019] ^A^	0.182 ± 0.148 [0.150] ^B^
Final	0.149 ± 0.165 [0.073]	0.047 ± 0.045 [0.040] ^A^	0.244 ± 0.180 [0.245] ^B^

Superscripts with different lowercase letters indicate differences between the baseline and the end of the study, and different uppercase letters indicate differences between fruit and vegetable groups.

## Supplementary Materials

The following are available online at https://www.mdpi.com/article/10.3390/nu13103614/s1, Table S1. Carotenoids dietary intake (µmol/day), Table S2. Contrast threshold at different degrees of visual angle, without and with glare (means ± SD).
