# Supplementary material for: Changes in Lutein Status Markers (Serum and Faecal Concentrations, Macular Pigment) in Response to a Lutein-Rich Fruit or Vegetable (Three Pieces/Day) Dietary Intervention in Normolipemic Subjects
Source: Nutrients. 2021 Oct 15;13(10):3614. doi: 10.3390/nu13103614 (PMC8538254; doi:10.3390/nu13103614)
Supplement: Supplementary file 1 [file nutrients-13-03614-s001.zip › nutrients-1385418-supplementary.pdf]

SUPPLEMENTARY MATERIAL

Table S1. Carotenoids dietary intake (μmol/day).

	Total sample (n=29)	Fruit group (n=14)	Vegetable group (n=15)
Lutein + zeaxanthin			
Basal	2.26 ± 1.31 [1.88] ^{a}	2.16 ± 1.03 [2.15]	2.35 ± 1.56 [1.88]
Final	4.42 ± 3.43 [3.45] ^{b}	6.06 ± 3.88 [4.18]^{A}	3.09 ± 1.99 [2.73]^{B}
β-cryptoxanthin			
Basal	1.25 ± 1.07 [0.93] ^{a}	1.52 ± 1.15 [1.73]	2.00 ± 0.97 [0.87]
Final	1.13 ± 1.04 [0.96] ^{b}	2.00 ± 0.78 [1.71]	0.33 ± 0.35 [1.71]
α-carotene			
Basal	0.89 ± 1.51 [0.40]	0.86 ± 1.80 [0.34]	0.92 ± 1.24 [0.40]
Final	0.50 ± 0.62 [0.19]	0.47 ± 0.48 [0.24]	0.56 ± 0.75 [0.20]
β-carotene			
Basal	4.00 ± 3.78 [2.58]	3.67 ± 4.54 [2.52]	4.30 ± 3.03 [3.88]
Final	3.83 ± 3.72 [2.71]	3.94 ± 2.96 [3.16]	4.00 ± 4.45 [2.44]
Lycopene			
Basal	20.21 ± 27.75 [5.97] ^{a}	3.04 ± 2.12 [2.61]^{A}	36.24 ± 31.07 [29.29]^{B}
Final	12.64 ± 14.94 [5.09] ^{b}	3.94 ± 1.92 [3.76]^{A}	22.25 ± 16.86 [20.11]^{B}

Different superscript letters indicate significant difference between basal and final concentrations (^{a} columns) and between groups (^{A} rows).

Difference between fruit and vegetable groups at baseline: lycopene (<0.001).

Differences between baseline and end of the study: a) total sample: lutein+zeaxanthin (0.002), β-cryptoxhantin (<0.001) and lycopene (0.009); b) between fruit and vegetable groups: lutein+zeaxanthin (0.012) and lycopene (<0.001)

Table S2. Contrast threshold at different degrees of visual angle, without and with glare (means ± SD).

Visual angle of the estimulus (°)	Total sample (n=29)	Fruit group (n=14)	Vegetable group (n=15)
Without glare			
6.3			
Basal	0.018 ± 0.013	0.020 ± 0.016	0.015 ± 0.008
Final	0.015 ± 0.007	0.016 ± 0.010	0.014 ± 0.005
4.0			
Basal	0.022 ± 0.013	0.025 ± 0.015	0.020 ± 0.012
Final	0.020 ± 0.011	0.020 ± 0.010	0.020 ± 0.012
2.5			
Basal	0.030 ± 0.018	0.033 ± 0.021	0.027± 0.015
Final	0.031 ± 0.019	0.033 ± 0.020	0.028 ± 0.018
1.6			
Basal	0.056 ± 0.034	0.057 ± 0.034	0.054 ± 0.034
Final	0.057 ± 0.043	0.063 ± 0.043	0.051 ± 0.043
1.0			
Basal	0.122 ± 0.083	0.125 ± 0.080	0.120 ± 0.090
Final	0.131 ± 0.092	0.145 ± 0.090	0.112 ± 0.094
0.7			
Basal	0.273 ± 0.156	0.280 ± 0.161	0.268 ± 0.154
Final	0.287 ± 0.150	0.296 ± 0.156	0.278 ± 0.146
With glare			
6.3			
Basal	0.014 ± 0.005	0.014 ± 0.005	0.013 ± 0.005
Final	0.015 ± 0.006	0.016 ± 0.007 [A]	0.013± 0.005 [B]
4.0			
Basal	0.022 ± 0.026	0.027 ±0.036	0.016 ± 0.008
Final	0.018 ± 0.010	0.020 ± 0.011 [A]	0.015 ± 0.008 [B]
2.5			
Basal	0.030 ± 0.015	0.028 ± 0.015	0.026 ± 0.015
Final	0.019 ± 0.017	0.032 ± 0.015	0.026 ± 0.018
1.6			
Basal	0.058 ± 0.039	0.061 ± 0.043	0.055 ± 0.035
Final	0.059 ± 0.040	0.059 ± 0.031	0.059 ± 0.048
1.0			
Basal	0.127 ± 0.082	0.124 ± 0.078	0.130 ± 0.090
Final	0.143 ± 0.106	0.155 ± 0.109	0.133 ± 0.104
0.7			
Basal	0.300 ± 0.106	0.290 ± 0.149	0.309 ± 0.139
Final	0.302 ± 0.162	0.295 ± 0.165	0.309 ± 0.029
